# The Advantage of Playing Home in NBA: Microscopic, Team-Specific and Evolving Features

**DOI:** 10.1371/journal.pone.0152440

**Published:** 2016-03-25

**Authors:** Haroldo V. Ribeiro, Satyam Mukherjee, Xiao Han T. Zeng

**Affiliations:** 1 Departamento de Física, Universidade Estadual de Maringá, Maringá, PR 87020-900, Brazil; 2 Northwestern Institute on Complex Systems, Northwestern University, Evanston, IL 60208, United States of America; 3 Indian Institute of Management, Udaipur, India; 4 Groupon, Inc. Chicago, IL 60654, United States of America; University of Maribor, SLOVENIA

## Abstract

The idea that the success rate of a team increases when playing home is broadly accepted and documented for a wide variety of sports. Investigations on the so-called “home advantage phenomenon” date back to the 70’s and ever since has attracted the attention of scholars and sport enthusiasts. These studies have been mainly focused on identifying the phenomenon and trying to correlate it with external factors such as crowd noise and referee bias. Much less is known about the effects of home advantage in the “microscopic” dynamics of the game (within the game) or possible team-specific and evolving features of this phenomenon. Here we present a detailed study of these previous features in the National Basketball Association (NBA). By analyzing play-by-play events of more than sixteen thousand games that span thirteen NBA seasons, we have found that home advantage affects the microscopic dynamics of the game by increasing the scoring rates and decreasing the time intervals between scores of teams playing home. We verified that these two features are different among the NBA teams, for instance, the scoring rate of the Cleveland Cavaliers team is increased ≈0.16 points per minute (on average the seasons 2004–05 to 2013–14) when playing home, whereas for the New Jersey Nets (now the Brooklyn Nets) this rate increases in only ≈0.04 points per minute. We further observed that these microscopic features have evolved over time in a non-trivial manner when analyzing the results team-by-team. However, after averaging over all teams some regularities emerge; in particular, we noticed that the average differences in the scoring rates and in the characteristic times (related to the time intervals between scores) have slightly decreased over time, suggesting a weakening of the phenomenon. This study thus adds evidence of the home advantage phenomenon and contributes to a deeper understanding of this effect over the course of games.

## Introduction

Competitive events among agents or groups are ubiquitous in nature and society. Understanding these competitive processes is a natural academic goal that finds important applications in economics, politics, and sports. In particular, sports are considered a natural laboratory for testing hypotheses and studying competitions [1, 2], with the tremendous advantage of offering more and more datasets that not only provide results or summaries of massive amounts of games, but also enable a complete recap of the within-game events of entire seasons of sport leagues. This unprecedented amount of data enabled scholars to probe patterns of such competitive events to a degree not before possible, answering many academic questions as well as elucidating sport folklores. Examples of such investigations include random walks or diffusive interpretations of the scoring process [3–12], discussions about the efficiency of sport competitions [4, 8, 13–17], analysis of player and team performance via networks tools [18–22] and tracking data [23], performance evolution in the Olympic Games [24], the role of coaching experience in the effective use of timeouts [25], reciprocity in passing patterns [26], cooperative play [27], Matthew effect in the longevity of careers in professional sport [28, 29], and the hot-hand phenomenon [10, 30–33].

Success in sport competitions is not only a fan demand, but also a long-standing business involving billions of dollars, management, finance, and marketing policies [34]. Thus, the identification of key factors that have a systematic influence on the success rate of teams and athletes goes beyond a theoretical question and may attract the interest of teams, coaches and players as well.

One of the consistent factors that are likely to affect the success rate in sport competitions is the game location. Despite some controversial findings, the idea that the success rate of a team (or a player) increases when playing home is widely accepted and documented for several sports. Starting with the seminal work of Schwartz and Stephen [35] in 1977, the “home advantage phenomenon” has motivated several investigations ever since [36–38]. A non-exhaustive list of sports where this phenomenon has been found include soccer [39–45], baseball [46, 47], ice hockey [48, 49], roller-hockey [50], basketball [51, 52], rugby [53], Australian football [54], water polo [55], volleyball [56], handball [57–59], and cricket [60]. This effect has also been observed in individual competitions of tennis [61, 62], golf [62], Winter Olympics sports [63], Summer Olympics sports [64], and several other individual sports [65]. Jamieson [66] reported an interesting meta-analysis on several sports (including some of the above-cited), where it was found that the overall home winning percentage is about 60%, and moderator factors such as time era (matches prior to the 50’s are more affected by this phenomenon than more recent ones) and sport (home advantage is more intense for soccer than several sports) were also identified. Researchers have also tried to assign causes related to the home advantage such as crowd noise [67, 68], audience hostility [69], away-team travels [70], tactics used by teams and coaches [71, 72], familiarity with the local playing facility [73] and referee bias [74] as well as pointed differences between teams from capital and inner cities [75].

Despite this considerable interest, much less is known about the effects of home advantage in the “microscopic” dynamics of games, that is, the changes this phenomenon causes within game events. Team-specific and evolving features of this phenomenon are other questions that are also rarely tackled in the previous pages. Exceptions include the works of Pollard and Pollard, which studied the evolution of the winning percentage at home for team sports [76] and regional variations in this percentage [77]. The former aspect along with a comparison between men and women soccer leagues was also discussed by Pollard and Gómez [78]. There are also evidence that home advantage is more intense at the beginning of basketball matches [51], time dependent for handball [58], and that the scoring processes is highly dynamic [16] for basketball. All these features raise several questions on the microscopic effects of the home advantage, and also on how these features may possibly differ among teams and time era.

In this article, we present a detailed study of the effects of home advantage in microscopic dynamics of more than sixteen thousand games spanning thirteen seasons of National Basketball Association (NBA). By analyzing the play-by-play events of the games, we find that the scoring rates increase when teams play home, whereas the time intervals between scores decrease. We have further observed that these features vary across teams, seasons, and game time (quarters). The overall average differences in the scoring rates and in the characteristic time intervals have slightly decreased over time, suggesting that home advantage has become weaker in the NBA. We also report a rank of the NBA teams according to the intensity of the home advantage in these two microscopic features of the game.

## Methods

### Data presentation

We have accessed data from the official web portal of ESPN, under the NBA section: http://espn.go.com/nba/. By browsing under the URL http://espn.go.com/nba/schedule/, we initially obtained all game identification between the years of 2001 and 2014. This game identification leads to a web page that contains information about the game, including game place and the play-by-play recap of game events (points, missing points, rebound, etc), see http://espn.go.com/nba/playbyplay?gameId=400489378 for an example of such pages for the game Detroit Pistols versus New York Knicks (playing home) in Jan 7, 2014. We thus downloaded all available game information pages, grouping the games according to the NBA season and removing special games such as NBA All-Star Games and matches involving foreign teams (Olympiacos, FC Barcelona, CSKA Moscow, etc). From these pages, we extracted the team names, game place, match date, and the score evolution *S*(*t*) of each team as a function of the game time *t*. At this stage, we further removed games for which no play-by-play events were available and also games in which the score evolution was not a monotonically increasing function of time *t*. These lead us to 16,133 games covering 13 NBA seasons (from the 2001–02 to the 2013–14 season). A small random sample of these data was further manually compared with the play-by-play events from the NBA official web page, and a perfect agreement was found for the score events. As these data are subject to updates, a snapshot has been provided as S1 Dataset.

## Results and Discussion

**Fig 1:**
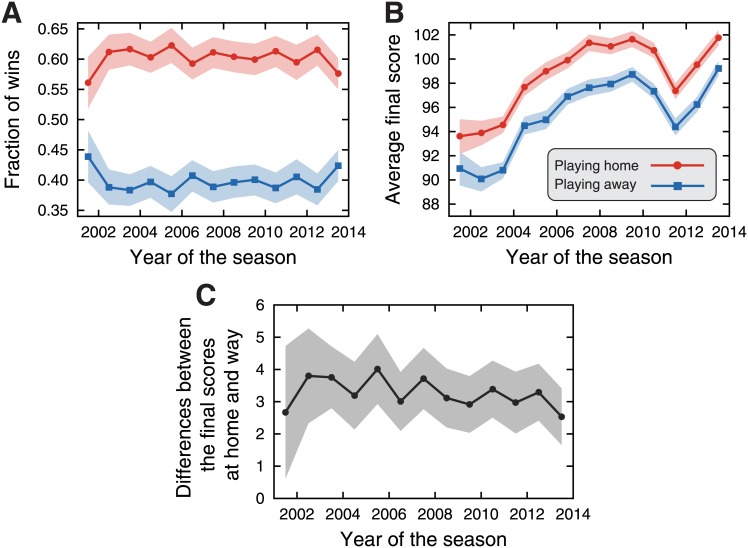
Macroscopic manifestation of the home advantage in NBA. (A) Average fraction of wins when playing home (red circles) and away (blue squares) along the thirteen NBA seasons studied here. (B) Average final score of the teams when playing home (red circles) and away (blue squares). (C) Evolution of the differences between the final scores and at home and away along the NBA seasons. In all plots, the shaded areas stand for 95% bootstrap confidence intervals.

We start by investigating the holistic idea of home advantage, that is, we ask whether the teams playing home have a large fraction of wins than those playing away. We calculate this fraction for each NBA season available in our dataset and the results are depicted in Fig 1A. We observe that teams playing home wins about 60% of the matches, 10% more than would be expected by chance. Similar values were reported for several basketball leagues [51, 52] and also in the meta analysis of Jamieson [66]. It is also worth noting that this fraction is almost constant over the years. Another manner of quantifying the macroscopic effect of home advantage is by evaluating the average final score of teams playing home and away. Fig 1B shows these quantities for each NBA season, where we (naturally) observe that teams playing home have greater final scores than those playing away. Interestingly, we notice that the average final scores show an evolving behavior: between seasons 2001–02 and 2009–10 these averages have increased, followed by a sharp decrease over the next two seasons (2010–11 and 2011–12) and again by an increasing behavior for the last two seasons, reaching almost the same values as in 2011–12. We have further investigated the difference between the final scores at home and away, as shown in Fig 1C. We observe that teams playing home score 3.3±0.1 points (average of all seasons) more than those playing away; also, this difference seems to present decreasing trend.

In order to quantify this trend, a linear regression model was adjusted to these data, yielding a weak decreasing trend of 0.08±0.03 points per year. It is worth noting that this regression does not account for team ability, which may introduce some bias in this evolving behavior. As discussed by Pollard and Gómez [78], more balanced leagues are more likely to be strongly affected by home advantage. Thus, this evolving trend could also be related to changes in the competitiveness of NBA seasons. However, studies have suggested that the competitive balance in NBA is stable over time and close to its average [14, 16] and that the scoring process is well described by a nearly unbiased random walk [9], suggesting that NBA teams are quite balanced. Furthermore, a decrease in the intensity of home advantage over time was also observed by Jamieson [66] for several sports. Therefore, despite the lack of a more precise approach for quantifying the evolving trend of home advantage (which is interesting but out of the scope of this article), our results are in agreement with recent findings on the subject and may be just slightly affected by the differences in ability among teams. Similar discussions apply to forthcoming analysis on evolving trends of other aspects of home advantage.

**Fig 2:**
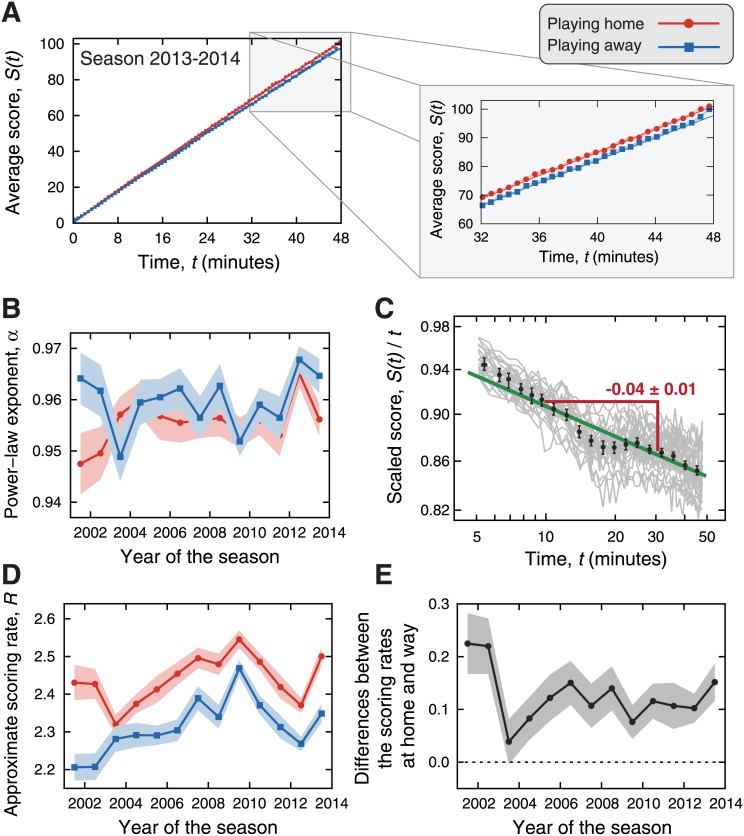
Evidence for home advantage in the score evolution. (A) Average score *S*(*t*) as a function of the game time *t* when playing home (red circles) and away (blue squares). These averages were calculated for the NBA season 2013–14 (see S1 Fig for all seasons). The last 16 minutes of the games are highlighted. The continuous lines (red for home and blue for away) represent the adjusted power-law models [*S*(*t*) = *Rt*^*α*^]. Notice that the difference between the average scores at home and away increases over time. (B) Evolution of the power-law exponent *α* over the NBA seasons. We observe practically no difference between playing home and away; however, the values of *α* are all smaller than one, indicating that the score evolution is slightly sub-linear. (C) The gray curves show the average score *S*(*t*) divided by *t* as a function of the game time *t* calculated for every NBA season and grouping the matches by field. The black dots are window average values over all curves and the error bars stand for 95% confidence intervals. The green line is a power-law fit to average tendency whose slope (power-law exponent) is 0.04±0.01. (D) Evolution of the approximate scoring rates *R* over the NBA seasons. The teams playing home display significantly larger rates (average over all seasons of 2.44±0.02 points per minute) than when playing away (2.31±0.02 points per minute). (E) Evolution of the differences between the scoring rates at home and away along the NBA seasons. Notice that these values display a decreasing tendency over the years. The shaded areas in the plots stand for 95% bootstrap confidence intervals.

The previous analysis has thus confirmed the existence of home advantage in the NBA games; however, it provides no clues on how this advantage emerge from the microscopic dynamics of the games. In order to investigate such aspects, we calculate the average score *S*(*t*) as a function of the game time *t*, after grouping the matches by seasons and field (home or away). Fig 2A shows an example of the behavior of *S*(*t*) for teams playing home and away in the season 2013–14. We observe that *S*(*t*) increases faster for teams playing home than those playing away and that a statistically significant difference appears around *t* ≈ 16 minutes. Similar behaviors are observed in all seasons (see S1 Fig).

Another intriguing feature of the behavior of *S*(*t*) is that it appears to increase as a nonlinear function of time, which is visible from its slight concave shape. To verify this nonlinear behavior, we have adjusted a linear function to the relationship log *S*(*t*) versus log *t*; in this case, a unitary linear coefficient indicates that *S*(*t*) increases linearly in time and deviations from the unitary value point out for a power-law behavior in *S*(*t*), that is,

$$S\left(t\right)=R\;t^{\alpha }\;,$$ (1)

with *α* being the power-law exponent (or the linear coefficient in the log-log relationship) and *R* a multiplicative constant (or the intercept in the log-log relationship). Fig 2B shows the values of *α* for playing home and away in each NBA season. We observe that these values are practically identical regarding playing home or away and that they can be well approximated by a constant plateau; however, we do observe that the values of *α* are all smaller than one, indicating that the scores increase (slightly) sub-linearly in time. Fig 2C shows the average score *S*(*t*) divided by *t* as a function of time for each season as well as for playing home and away. If the average score was linear in time, these curves would be approximated by horizontal lines in these log-log plots; instead, we observe a decreasing behavior. Furthermore, by fitting a power-law function to the average behavior of *S*(*t*)/*t* versus *t*, we find that the power-law exponent is 0.04±0.01, a value that is consistent with the values of *α* reported in Fig 2B, that is, for *S*(*t*)∼*t*^*α*^, we expect *S*(*t*)/*t* ∼ *t*^*α*−1^.

The sub-linear behavior of the scores versus time indicates the scoring rates are not constant over time, as was also observed by Gabel and Redner [9]; actually, the values of *α* < 1 indicate that the scoring rates decrease with the passing of time—a fact probably related to the physical wear of the athletes along the game. However, the values of *α* are not very different from one, and we may consider that the value of *R* represents an approximate the scoring rate. Fig 2D shows the values of *R* estimated for teams playing home and away for every NBA season. We notice that teams playing home have a statistically significantly larger scoring rate than those playing away. By averaging over all seasons in our dataset, we estimate that teams score at *R* = 2.44±0.02 points per minute at home, whereas *R* = 2.31±0.02 points per minute is the average scoring rate in away matches. We further calculate the difference between the values of *R* at home and away, as shown in Fig 2E. Despite the sharp change occurred in the 2002–03 season and similarly to the results of Fig 1, the differences between the values of *R* seems to decrease over time. A linear regression model adjusted to these data indicates that difference in the scoring rates is diminishing at a slight pace of 0.004±0.002 points per minute per year.

**Fig 3:**
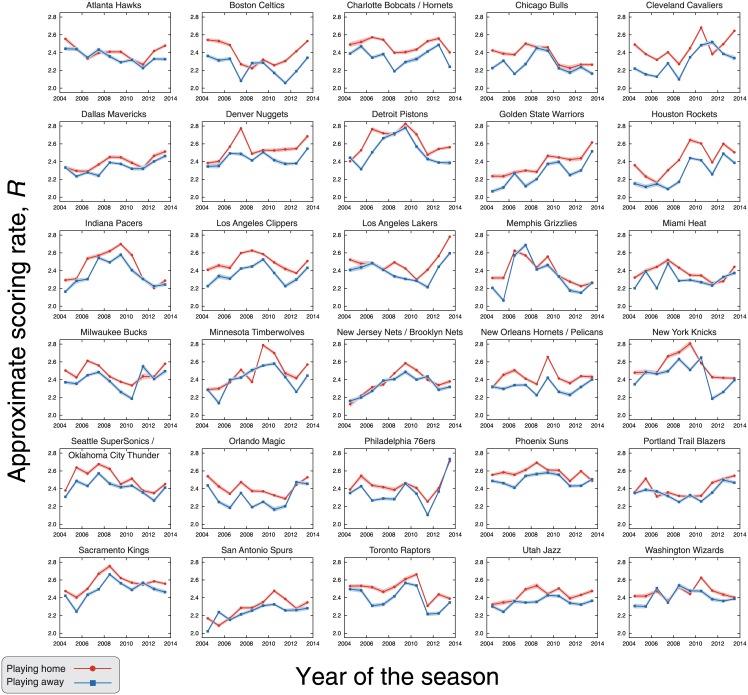
Evolution of the scoring rate when playing home and away for each NBA team. The panels show the approximate scoring rates *R* when playing home (red circles) and away (blue squares) for every team and season from 2004–05 to 2013–14, period in which the teams were the same. The shaded areas are 95% bootstrap confidence intervals. Notice that the scoring rates are systematically larger when the team plays home; however, we do observe some inversions and that values of *R* vary among teams and seasons.

**Fig 4:**
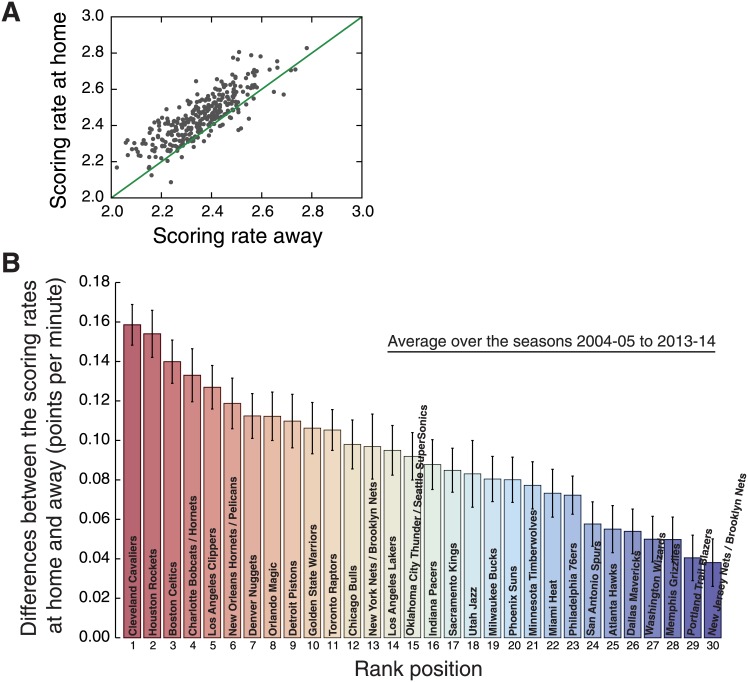
Ranking NBA teams according to the difference between the scoring rate at home and away. (A) Scoring rate at home versus scoring rate away (that is, the values of *R*). The dots represent the scoring rates (at home and away) for every team and NBA season, and the green line is a linear function (with a unitary linear coefficient and a zero intercept). Notice that there are only a few cases in which the scoring rate is larger when playing away than when playing home. (B) Average of the difference between the scoring rates at home and away for each NBA team (in descending order). These averages were calculated over the seasons 2004–05 to 2013–14 (during this period the teams were the same; see and S6 Fig for all scoring rates) and the error bars are 95% bootstrap confidence intervals.

The approximate scoring rate *R* previously described is an average over all teams. This begs the intriguing question whether the values of *R* differs from team to team. To address this question, we estimate the values of *R* at home and away for each NBA team between the seasons 2004–05 and 2013–14. In this period, the number of teams was fixed as thirty (current number) and the same teams competed in the league. The only change in the list of teams occurred at the end of the 2007–08 season, when the team Seattle SuperSonics was relocated to Oklahoma City and now plays as the Oklahoma City Thunder. We have considered this event only as a name change and assumed the matches before and after the 2007–08 are from the same team. In order to estimate the values of *R* for each team and focus only on its evolution, we have fixed the values of *α* to its overall average value when fitting the relationships between log *S*(*t*) and *t* (Eq 1). Fig 3 shows the values of *R* for each team and season. Despite a few exceptions, we observe that the scoring rates are systematically larger when playing home than away for all teams. In Fig 4A, we plot the values of *R* at home against the values of the *R* estimated for away matches for each team and season, where it is further evident that the occurrence of teams which larger scoring rates in away matches in a NBA season is very rare (around 1% of the teams by season).

We further observe that despite the complicated evolving behavior of *R* reported in Fig 3 for each team, one can note that teams such as New Jersey Nets (now the Brooklyn Nets) and Portland Trail Blazers have small differences between the scoring rates at home and away when compared with other teams such as Cleveland Cavaliers and Houston Rockets. To quantify these differences, we have estimated the scoring rates at home and away from the evolution scores *S*(*t*) averaged over the seasons 2004–05 to 2013–14 for each NBA team (see S2 Fig). Fig 4B shows a rank of the teams according to the difference between the scoring rates at home and away and confirms the existence of statistically significant differences among them. For instance, Cleveland and Houston have the largest differences and score about 0.16 point per minute more when playing home, whereas New Jersey and Portland have the smallest differences (around 0.04 point per minute more in home matches).

**Fig 5:**
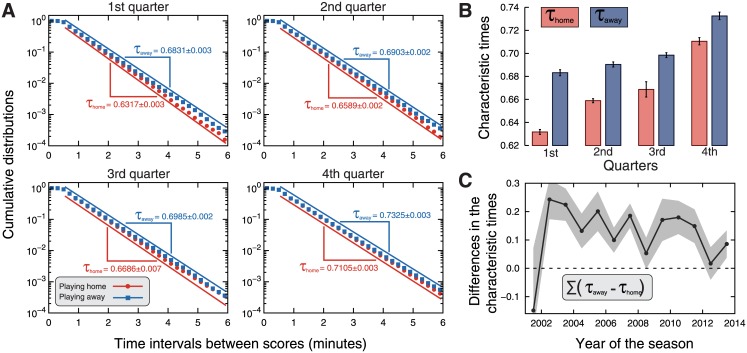
Evidence for home advantage in the time intervals between scores. (A) Cumulative distributions of the time intervals between stores when the teams play home (red dots) and away (blue squares). The panels show the distributions for the four quarters (period of 12 minutes in which the games are played). Here we have aggregated data from all seasons (see S3, S4, S5 and S6 Figs for individual results). All distributions are well approximated by exponential distributions, that is, *P*(Δ*t*)∼*e*^−Δ*t*/*τ*^, where *τ* = *τ*_home_ is the characteristic time interval when playing home and *τ* = *τ*_away_ is the analogous when playing away. Notice that the plots are in log-lin scale and thus the exponential decay is linearized. The values of *τ*_home_ and *τ*_away_ were estimated via maximum likelihood method and are shown in the plots. The straight lines are guides for the eyes indicating the adjusted behavior of *P*(Δ*t*). We observe that these distributions decay faster for teams playing home than playing away. (B) Bar plots of the characteristic times *τ*_home_ and *τ*_away_ for each quarter. The error bars stand for 95% bootstrap confidence intervals. The characteristic times are systematically smaller when the teams play home than when playing way; we further observe that the difference *τ*_away_ − *τ*_home_ decreases with the passing of the quarters. (C) Evolution of the sum of the differences between the characteristic times at home and away [∑(*τ*_away_ − *τ*_home_), over all quarters] along the NBA seasons. The shaded areas stand for 95% bootstrap confidence intervals.

We now focus on quantifying the role of playing home in another microscopic game feature: the time intervals between scores. In this context, it is natural to imagine that teams playing home may display a faster rhythm, perhaps driven by the home team crowd [40, 68, 69]. In order to investigate this possibility, we have estimated the probability distributions of the time intervals between scores in each quarter of the game. Fig 5A shows these distributions when aggregating data from all seasons and grouping home and away matches. We note that these empirical distributions are well described by exponential distributions, that is,

P(Δt)=(1/τ)exp(-Δt/τ), (2)

where *P*(*t*) is the probability of finding a time interval between scores equal to Δ*t* and *τ* is the characteristic time interval (the only distribution parameter). Similar exponential distributions were reported by Gabel and Redner [9] when considering all quarters together. In addition, we observe that the exponential decays of these distributions are faster for the time intervals occurring in home matches than in away matches. To quantify this difference, we estimate (via maximum likelihood method) the values of the characteristic time interval *τ* when playing home (*τ* = *τ*_home_) and away (*τ* = *τ*_away_) for each quarter. The values of these parameters are shown in Fig 5A as well as are represented in a bar plot in Fig 5B. We find that the characteristic time interval between scores is statistically significant smaller in home matches than in away matches in all quarters. It is worth noting that the values of *τ*_home_ and *τ*_away_ are also the average value of the time intervals Δ*t*; thus, home teams actually have a faster scoring pace. We observe that both the values of *τ*_home_ and *τ*_away_ increase along the quarters of the game; again, a fact that is likely caused by the fatigue process of the players. However, we do notice that the largest gap between *τ*_home_ and *τ*_away_ occurs in the first quarter and that this gap is gradually reduced as the game progresses. This result is in agreement with findings of Jones [51] and suggests that this aspect of the home advantage is stronger at the beginning of the matches, which could be caused by the usual intense reception of the home team by its fans or also by the initial unfamiliarity of the guest team with the arena and its audience.

We have also evaluated the probability distributions of the time intervals between scores after grouping our data by NBA season. We show these distribution for each game quarter in S3, S4, S5 and S6 Figs, where similar exponential behaviors also emerge. Once again, we estimate the parameters *τ*_home_ and *τ*_away_ via maximum likelihood method for each quarter and season. Fig 5C shows the evolution of the sum of the differences between *τ*_away_ and *τ*_home_ over the quarters. Despite the some fluctuations and similarly to our previous results (Figs 1C and 2E), the differences in the characteristic time intervals between scores appear to decrease over time. A linear regression on this trend (after discarding the season 2001–02) indicates a decreasing tendency of 0.012±0.005 minutes per year.

**Fig 6:**
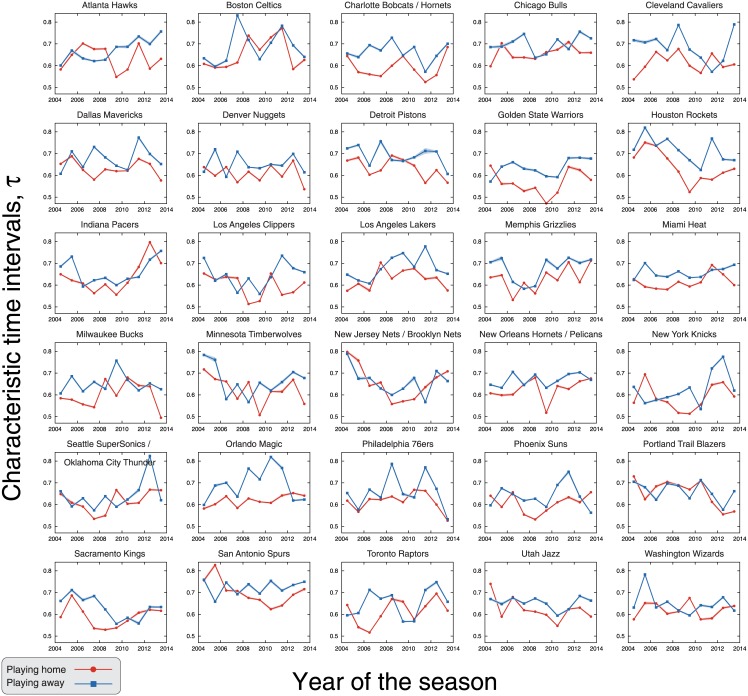
Evolution of the characteristic time intervals when playing home and away for each NBA team. The panels show the characteristic time intervals between scores when playing home (*τ*_home_, red circles) and away (*τ*_away_, blue squares) for every team and season from 2004–05 to 2013–14, period in which the teams were the same. Here we have aggregated data from all quarters and estimated the characteristic times via maximum likelihood method. The shaded areas are 95% bootstrap confidence intervals. Notice that the characteristic times are systematically larger when the team plays away; still, we note some inversions and that the values vary among teams and seasons.

**Fig 7:**
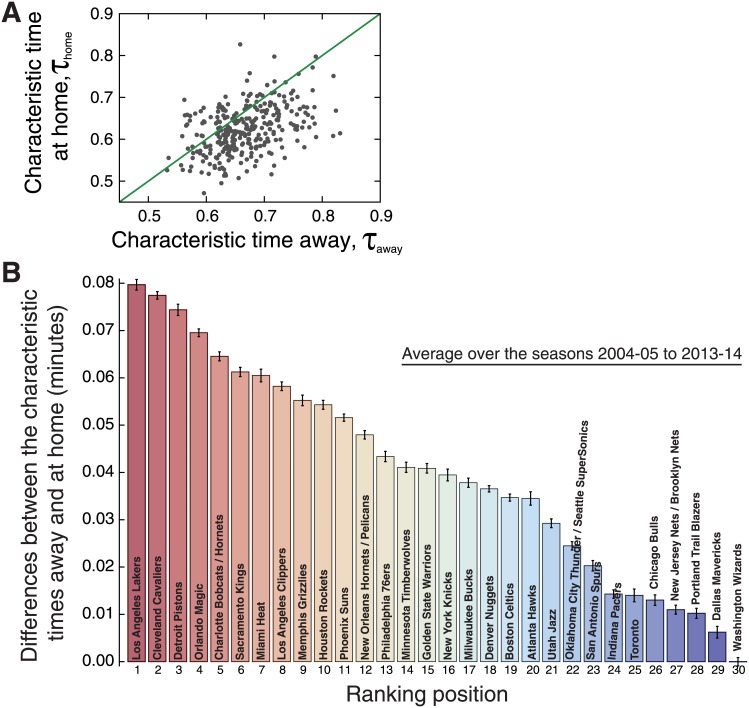
Ranking NBA teams according to the difference between the characteristic time intervals at home and away. (A) Characteristic time intervals between scores at home (*τ*_home_) versus away (*τ*_away_). The dots represent the values of the characteristic times (at home and away) for every team and season from 2004–05 to 2013–14 (during this period the teams were the same), and the green line is a linear function (*τ*_home_ = *τ*_away_). Here we have aggregated data from all quarters and estimated the characteristic times via maximum likelihood method. Notice that there are only a few cases in which the characteristic time is larger when playing home than when playing away. (B) Average of the difference between *τ*_away_ and *τ*_home_ for each NBA team (in descending order). These averages were calculated over the seasons 2004–05 to 2013–14 (see S7 Fig for the cumulative distributions) and the error bars are 95% bootstrap confidence intervals.

Finally, we turn our attention to possible team-specific features related to the time intervals between scores. To do so, we proceed as it was did for the scoring rates, that is, we have estimated the values of the characteristic times (*τ*_home_ and *τ*_away_) for each of the thirty teams that have competed in the league during the seasons 2004–05 and 2013–14. Different from the previous analysis, we have now aggregated data from all quarters for obtaining a more reliable estimate of the time intervals distribution and its parameter. Fig 6 shows the values of the characteristic times for playing home and away (estimated via maximum likelihood method) for each team and season. We observe that values of *τ* are (despite some inversions) systematically larger when the teams play away from home, that is, the average time interval between scores is larger in away matches. Fig 7A shows a scatter plot of the values of *τ*_home_ versus *τ*_away_ for every team and season, where we observe that the occurrence of teams with larger characteristic times at home in a season is very rare (around 2% of the teams by season). Despite that, we notice that this relationship exhibits larger fluctuations when compared with Fig 4A. However, the linear regression *τ*_home_ = *b* + *τ*_away_ finds *b* = −0.043±0.004 (*p*-value <10^−16^), indicating that the occurrence of *τ*_home_ < *τ*_away_ cannot be explained by chance.

Similarly to the case of the scoring rates, the evolving behavior of the characteristic times reported in Fig 6 is somehow a noisy one (visually larger than that reported in Fig 3). Part of this behavior could be associated with the intrinsic changes of the teams; however, there may also be some fluctuations related to the different number of events employed when estimating values of the characteristic times. Thus, just as it was did when ranking the teams according to the scoring rates, we have aggregated the time interval events of the teams over the seasons 2004–05 to 2013–14 for estimating the distributions *P*(Δ*t*) as well as the parameters *τ*_home_ and *τ*_away_ (see S7 Fig). Fig 7B shows a rank of the teams according to the difference *τ*_away_ − *τ*_home_, where we observe statistically significant difference among teams. This difference ranges from about 0.08 minutes for the Los Angeles Lakers and Cleveland to around zero for the Washington. It is worth noting that the ranking based on the scoring rates (Fig 4B) and on the characteristic times are not the same, that is, there are several disagreements between the two rankings. However, they are correlated as measured by the Spearman’s rank correlation coefficient (*ρ* = 0.50, *p*-value = 0.005) or by the Kendall’s one [79] (*ρ* = 0.34, *p*-value = 0.009), suggesting that teams playing home not only have higher scores but also score at a faster rate.

## Conclusions

In this work, we presented a new view of the home advantage phenomenon by focusing on the microscopic features of NBA matches. Specifically, we asked about the role of playing home on the dynamics of the score events within NBA games. Firstly, we studied the time behavior of the scores along the games, where it was found that the average score increases slightly sub-linearly in time for home and away matches. Based on this behavior, we defined an approximate scoring rate and determined that teams score an average of 0.13 points per minute more in home matches. We also verified that this number appears to be diminishing over the seasons at a slight pace, a behavior that also appears in other sports [66]. We further estimated the scoring rates at home and away for every team and season (from 2004–05 to 2013–14), where we observed that the difference between these rates changes from team to team. A ranking of teams according to the difference between the scoring rates at home and away was also presented. Next, we focused our attention on the time intervals between scores. The probability distribution of these times was found to be in good agreement with an exponential distribution, where the characteristic times for away matches are larger than the values for home matches. We noticed that this gap is gradually reduced over the game progress (that is, along the game quarters), which indicates that home advantage in NBA is mostly accumulated in the beginning of the matches, as was also discussed by Jones [51]. In addition, the difference in the characteristic times has decreased over the NBA seasons. Analogous to the scoring rates, the difference in the characteristic times at home and away is a team-specific feature, which enabled us to rank the teams according to this difference. Both the reduction in the difference between the scoring rates and in the characteristic time intervals suggest that the microscopic effect of the home advantage phenomenon is slowly becoming weaker. This change might be because teams are using better strategies to overcome disadvantages when playing away.

Our thus work provides new clues about the role of playing home and away in sport competitions by showing how two microscopic features of NBA games are affected. We further believe that our approach could be useful for a better understanding of the universality of home advantage across different sports; in particular, it may help to differentiate this phenomenon between interdependent sports such as basketball or soccer (where teams members need more cooperation to complete tasks) and independent ones such as baseball.

## Supporting Information

S1 Dataset Dataset employed in this study. Each line of the file corresponds to a game. The lines are formatted as follows:
{{{time, score},…,{time, score}},{{time, score},…,{time, score}},{{time, score},…,{time, score}},{{time, score},…,{time, score}},}{{{time, score},…,{time, score}},{{time, score},…,{time, score}},{{time, score},…,{time, score}},{{time, score},…,{time, score}},}{{“team playing home”, “team playing away”},{Final score of team playing home, Final score of team playing away},{ “game year”, “game month”}}
The first set of brackets represent the evolution of the score for a team playing home and the second one is the same for a team playing away. The inner brackets correspond to each NBA quarter.
(GZ) Click here for additional data file.

S1 Fig Average score *S*(*t*) as function of the game time *t* when playing at home (red circles) and away (blue squares). Each panel shows the results for a NBA season (indicated in the plots) and the continuous lines (red for home and blue for away) represent the adjusted power-law models [*S*(*t*) = *Rt*^*α*^].
(PDF)

S2 Fig Average score *S*(*t*) as function of the game time *t* when playing home (red circles) and away (blue squares) for each NBA team along the seasons 2004–05 to 2013–14. Each panel shows the results for a NBA team (indicated in the plots) and the continuous lines (red for home and blue for away) represent the adjusted power-law models [*S*(*t*) = *Rt*^*α*^].
(PDF)

S3 Fig Cumulative distributions of the time intervals between stores when the teams play home (red dots) and away (blue squares) for the first quarter of the games. The panels show the distributions in log-lin scale for each NBA season. The straight lines are guides for the eyes indicating the adjusted exponential behavior of these distributions.
(PDF)

S4 Fig Cumulative distributions of the time intervals between stores when the teams play home (red dots) and away (blue squares) for the second quarter of the games. The panels show the distributions in log-lin scale for each NBA season. The straight lines are guides for the eyes indicating the adjusted exponential behavior of these distributions.
(PDF)

S5 Fig Cumulative distributions of the time intervals between stores when the teams play home (red dots) and away (blue squares) for the third quarter of the games. The panels show the distributions in log-lin scale for each NBA season. The straight lines are guides for the eyes indicating the adjusted exponential behavior of these distributions.
(PDF)

S6 Fig Cumulative distributions of the time intervals between stores when the teams play home (red dots) and away (blue squares) for the fourth quarter of the games. The panels show the distributions in log-lin scale for each NBA season. The straight lines are guides for the eyes indicating the adjusted exponential behavior of these distributions.
(PDF)

S7 Fig Cumulative distributions of the time intervals between stores when the teams play home (red dots) and away (blue squares) for each NBA team along the seasons 2004–05 to 2013–14. The dashed lines are guides for the eyes indicating the adjusted exponential behavior of these distributions.
(PDF)
