# Supplementary figures and images for: The Advantage of Playing Home in NBA: Microscopic, Team-Specific and Evolving Features
Source: PLoS One. 2016 Mar 25;11(3):e0152440. doi: 10.1371/journal.pone.0152440 (PMC4807825; doi:10.1371/journal.pone.0152440)

Average score

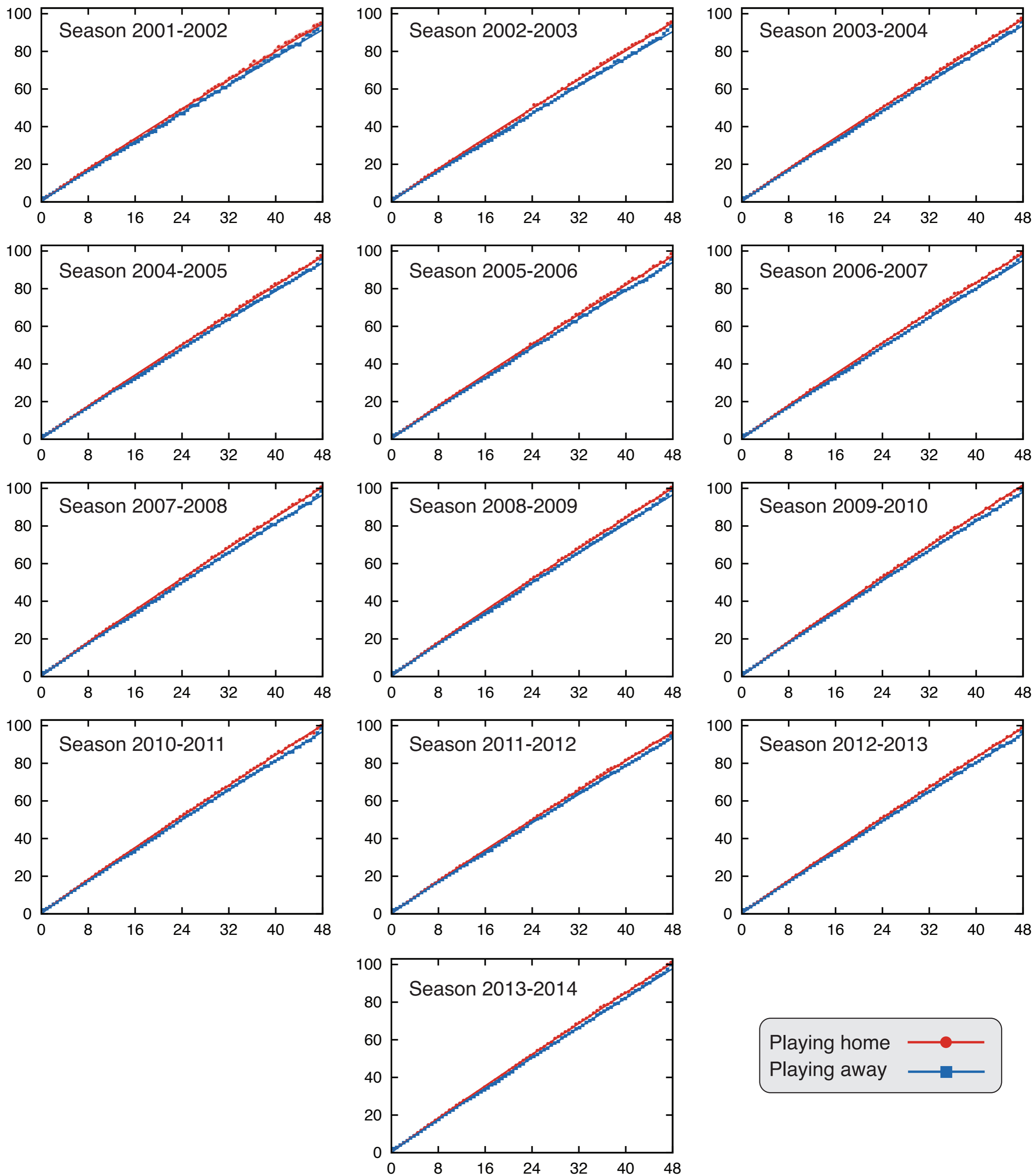

Time (minutes)

Playing home

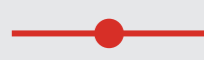

Playing away

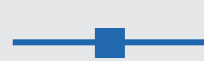

Supplement: S1 Fig — Each panel shows the results for a NBA season (indicated in the plots) and the continuous lines (red for home and blue for away) represent the adjusted power-law models [S(t) = Rtα]. (PDF) [file pone.0152440.s002.pdf]

# Seasons 2004-05 to 2013-14

Average score,  $S(t)$

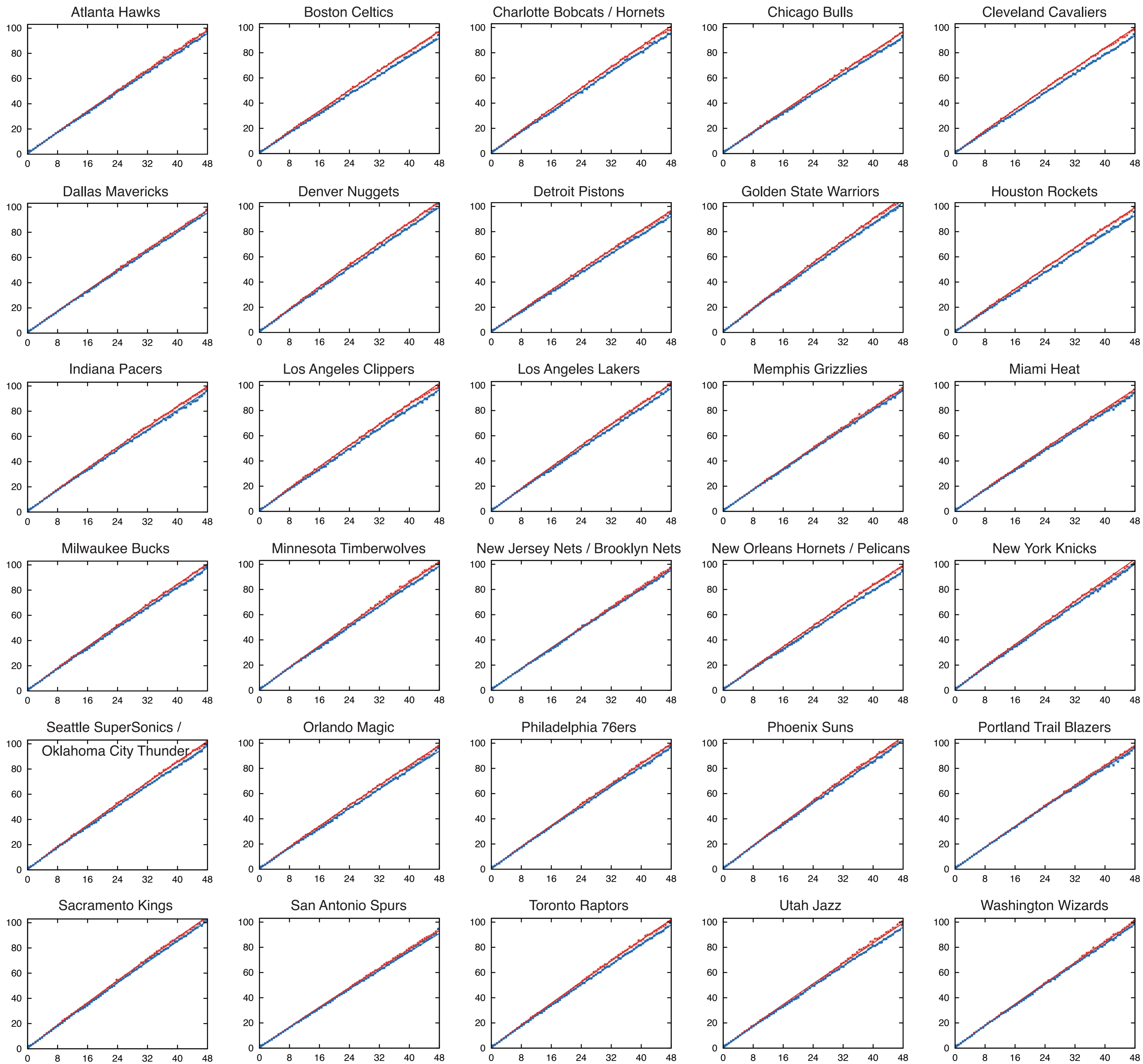

Playing home —●—  
Playing away —■—

Time,  $t$  (minutes)

Supplement: S2 Fig — Each panel shows the results for a NBA team (indicated in the plots) and the continuous lines (red for home and blue for away) represent the adjusted power-law models [S(t) = Rtα]. (PDF) [file pone.0152440.s003.pdf]

# First quarter

Cumulative distributions

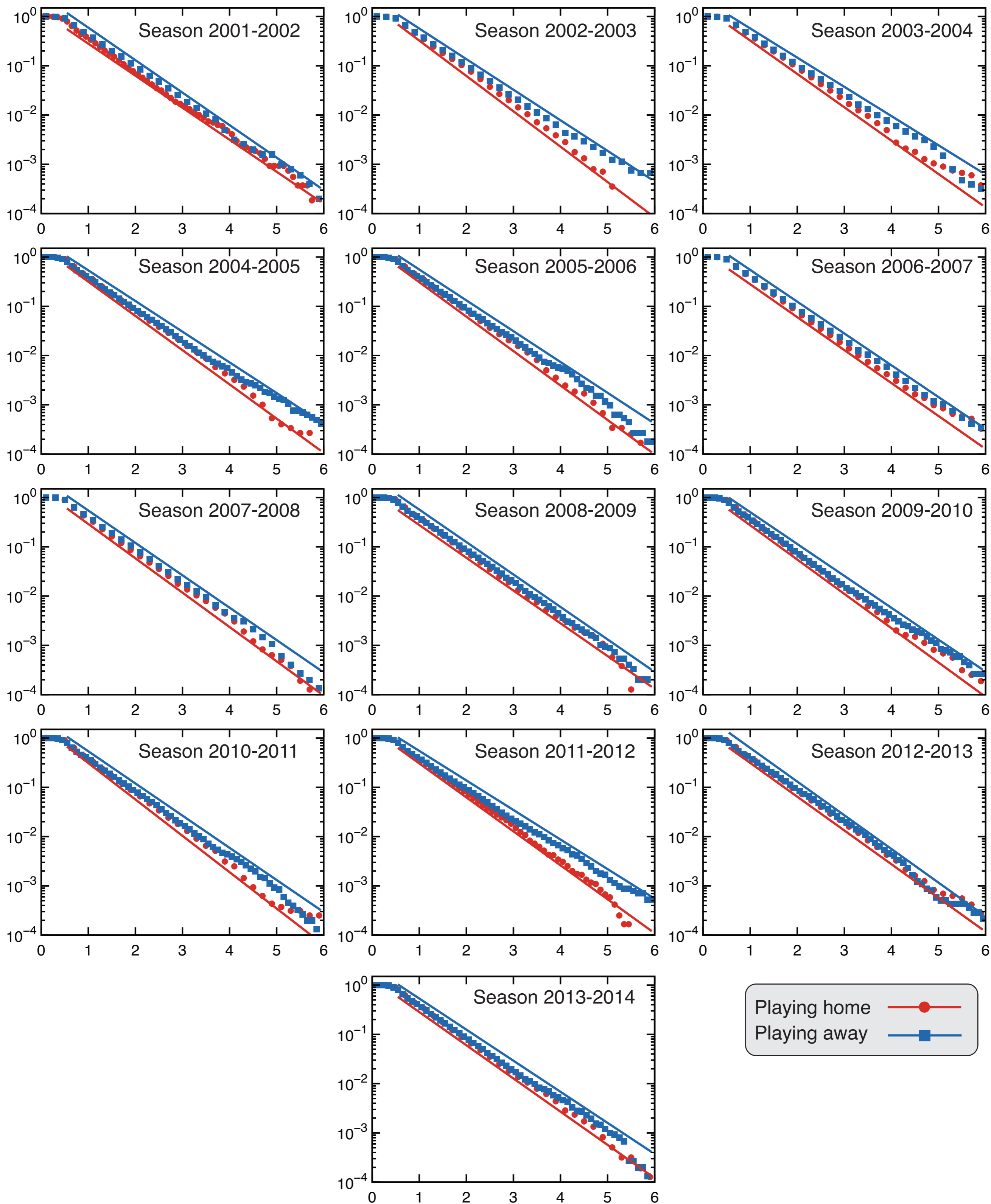

Time intervals between scores (minutes)

Supplement: S3 Fig — The panels show the distributions in log-lin scale for each NBA season. The straight lines are guides for the eyes indicating the adjusted exponential behavior of these distributions. (PDF) [file pone.0152440.s004.pdf]

# Second quarter

Cumulative distributions

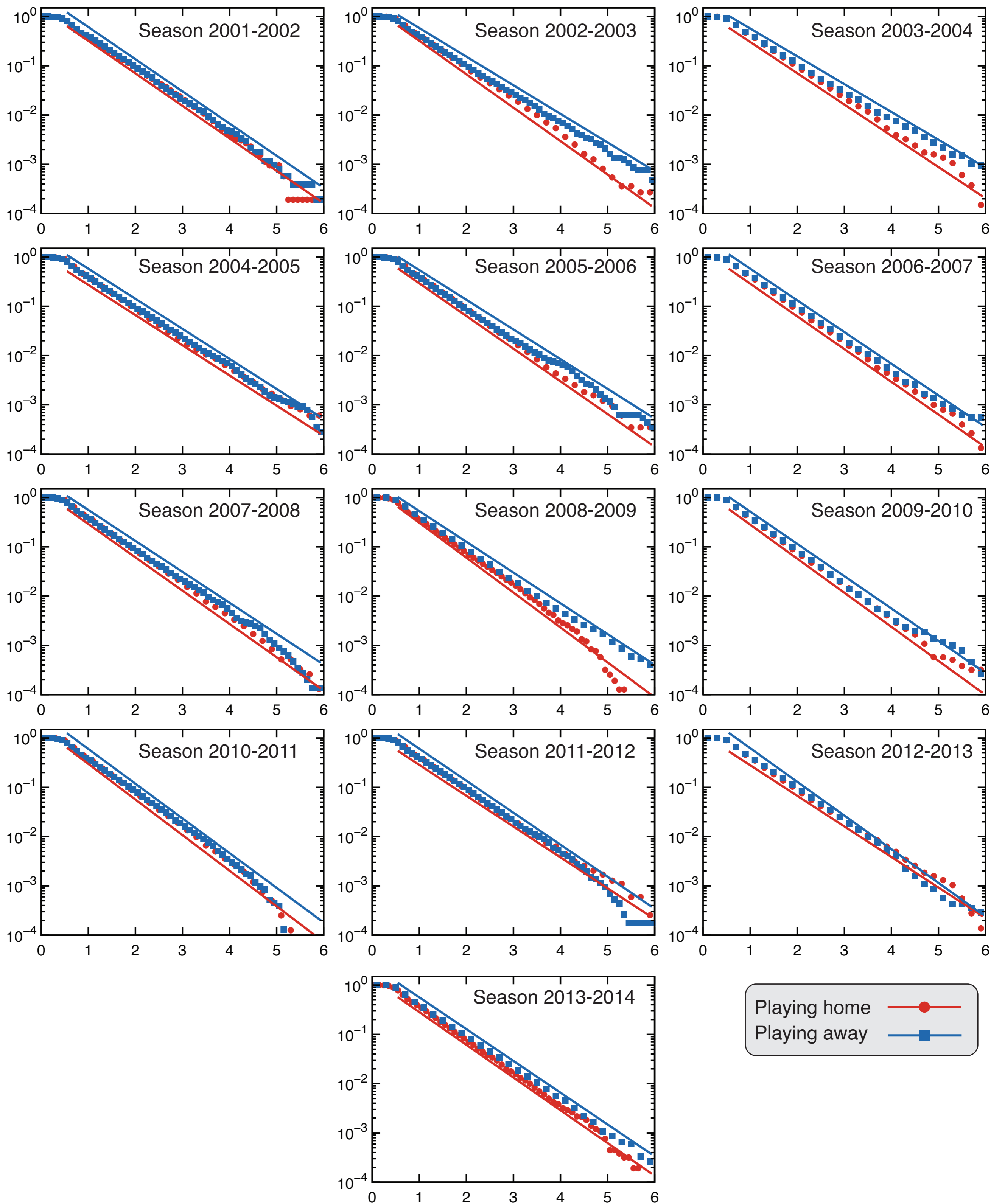

Time intervals between scores (minutes)

Supplement: S4 Fig — The panels show the distributions in log-lin scale for each NBA season. The straight lines are guides for the eyes indicating the adjusted exponential behavior of these distributions. (PDF) [file pone.0152440.s005.pdf]

# Third quarter

Cumulative distributions

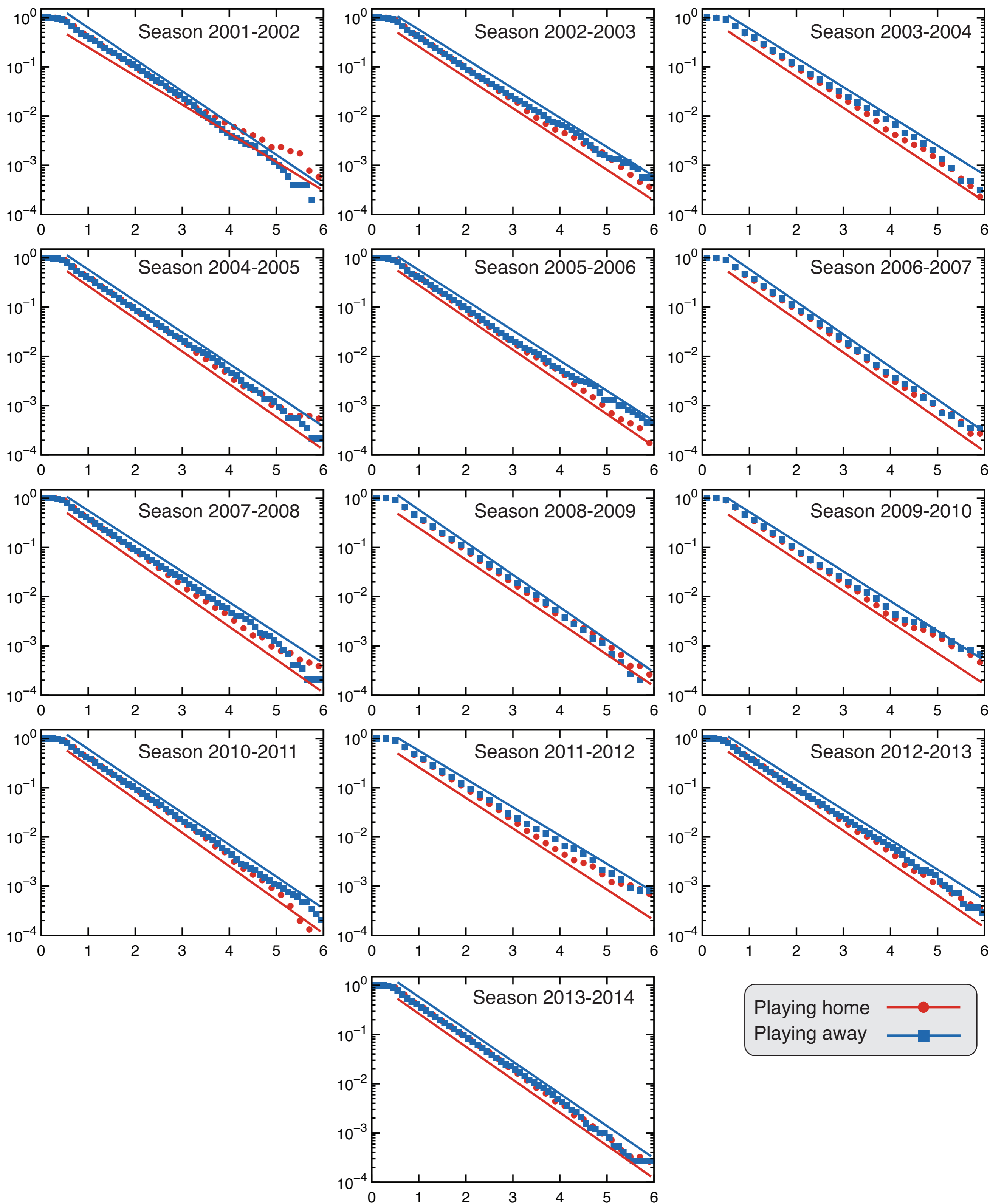

Time intervals between scores (minutes)

Supplement: S5 Fig — The panels show the distributions in log-lin scale for each NBA season. The straight lines are guides for the eyes indicating the adjusted exponential behavior of these distributions. (PDF) [file pone.0152440.s006.pdf]

# Fourth quarter

Cumulative distributions

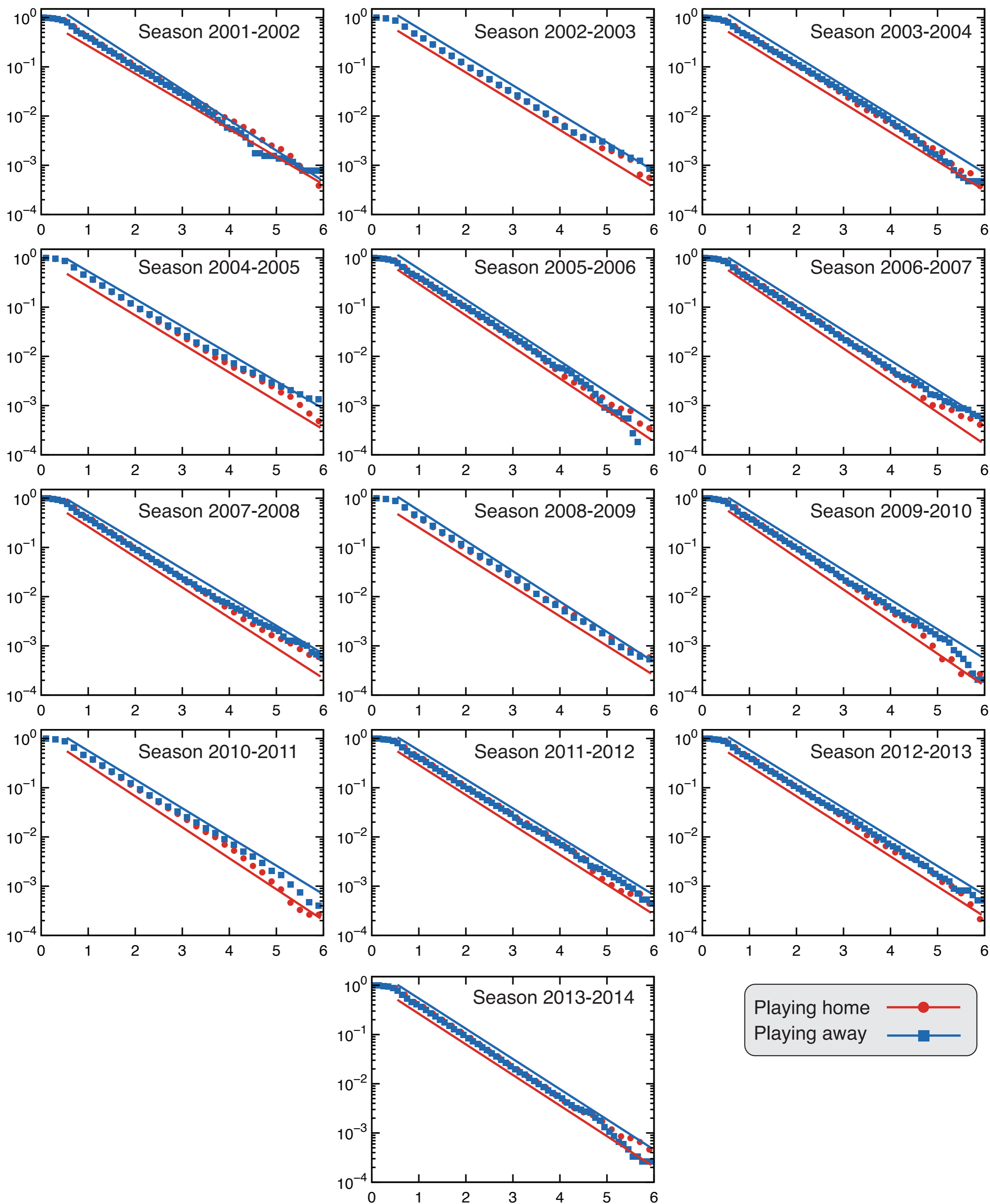

Time intervals between scores (minutes)

Supplement: S6 Fig — The panels show the distributions in log-lin scale for each NBA season. The straight lines are guides for the eyes indicating the adjusted exponential behavior of these distributions. (PDF) [file pone.0152440.s007.pdf]
