# Supplementary material for: The Advantage of Playing Home in NBA: Microscopic, Team-Specific and Evolving Features
Source: PLoS One. 2016 Mar 25;11(3):e0152440. doi: 10.1371/journal.pone.0152440 (PMC4807825; doi:10.1371/journal.pone.0152440)
Supplement: S7 Fig — The dashed lines are guides for the eyes indicating the adjusted exponential behavior of these distributions. (PDF) [file pone.0152440.s008.pdf]

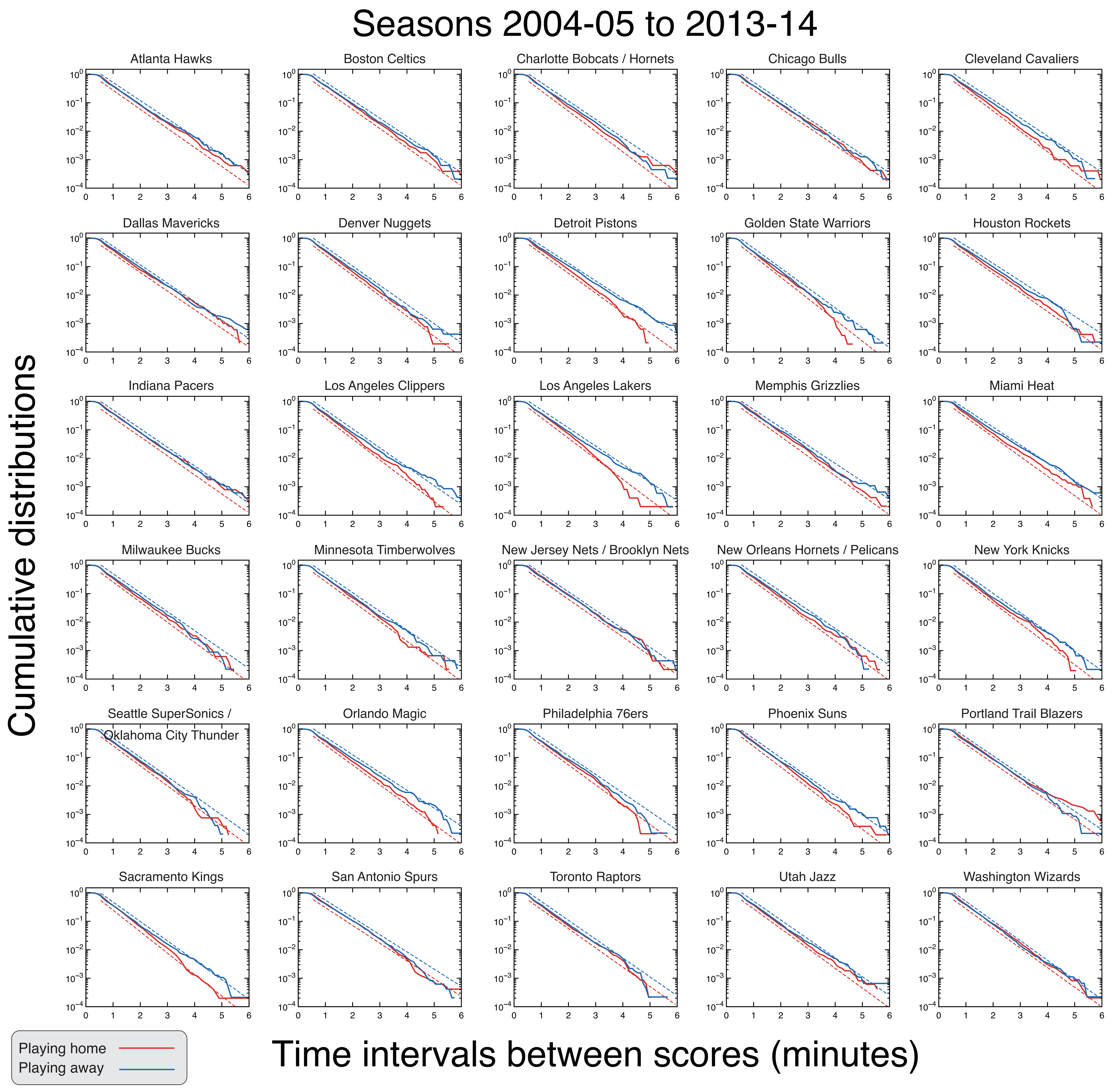
Seasons 2004-05 to 2013-14
Atlanta Hawks
Boston Celtics
Charlotte Bobcats / Hornets
Chicago Bulls
Cleveland Cavaliers
Dallas Mavericks
Denver Nuggets
Detroit Pistons
Golden State Warriors
Houston Rockets
Indiana Pacers
Los Angeles Clippers
Los Angeles Lakers
Memphis Grizzlies
Miami Heat
Milwaukee Bucks
Minnesota Timberwolves
New Jersey Nets / Brooklyn Nets
New Orleans Hornets / Pelicans
New York Knicks
Seattle SuperSonics / Oklahoma City Thunder
Orlando Magic
Philadelphia 76ers
Phoenix Suns
Portland Trail Blazers
Sacramento Kings
San Antonio Spurs
Toronto Raptors
Utah Jazz
Washington Wizards
Cumulative distributions
Time intervals between scores (minutes)
Playing home
Playing away
